# New insights into the biomimetic design and biomedical applications of bioengineered bone microenvironments

**DOI:** 10.1063/5.0065152

**Published:** 2021-11-03

**Authors:** Cláudia S. Oliveira, Sander Leeuwenburgh, João F. Mano

**Affiliations:** 1 Department of Chemistry, CICECO–Aveiro Institute of Materials, University of Aveiro, Campus Universitário de Santiago, 3810-193 Aveiro, Portugal; 2 Department of Dentistry-Regenerative Biomaterials, Radboud University Medical Center, Radboud Institute for Molecular Life Sciences, Philips van Leydenlaan 25, 6525 EX Nijmegen, The Netherlands

## Abstract

The bone microenvironment is characterized by an intricate interplay between cellular and noncellular components, which controls bone remodeling and repair. Its highly hierarchical architecture and dynamic composition provide a unique microenvironment as source of inspiration for the design of a wide variety of bone tissue engineering strategies. To overcome current limitations associated with the gold standard for the treatment of bone fractures and defects, bioengineered bone microenvironments have the potential to orchestrate the process of bone regeneration in a self-regulated manner. However, successful approaches require a strategic combination of osteogenic, vasculogenic, and immunomodulatory factors through a synergic coordination between bone cells, bone-forming factors, and biomaterials. Herein, we provide an overview of (i) current three-dimensional strategies that mimic the bone microenvironment and (ii) potential applications of bioengineered microenvironments. These strategies range from simple to highly complex, aiming to recreate the architecture and spatial organization of cell–cell, cell-matrix, and cell-soluble factor interactions resembling the *in vivo* microenvironment. While several bone microenvironment-mimicking strategies with biophysical and biochemical cues have been proposed, approaches that exploit the ability of the cells to self-organize into microenvironments with a high regenerative capacity should become a top priority in the design of strategies toward bone regeneration. These miniaturized bone platforms may recapitulate key characteristics of the bone regenerative process and hold great promise to provide new treatment concepts for the next generation of bone implants.

## I. INTRODUCTION

The self-healing capacity of bone can become significantly compromised in the case of, e.g., large bone defects, patient co-morbidities, aging, and inflammatory disorders.^1^ These factors complicate the natural process of bone regeneration. Consequently, osseous reconstructive surgery is often still a major clinical challenge for the treatment of severe fractures and/or large bone defects. Even the gold standard treatment, i.e., the use of autologous bone grafts, has been associated with poor bone regeneration, infection, and limited availability.^2^ Aiming to circumvent the limitations of these conventional medical procedures, bone tissue engineering (BTE) and cell-based therapies have emerged as an alternative to engineer bone tissue for implantation. In fact, one of the most promising strategies entails mimicry of the human bone microenvironment under *in vitro* conditions to provide cells the structural, biochemical, and/or instructive signals that positively influence bone tissue regeneration and healing after implantation.

Presently, the ever-increasing knowledge of the physiology, architecture, composition, macro/microscopic properties, and mechanisms of formation and repair of bone^3–5^ has inspired the design of a plethora of biomimetic bone microenvironments, ranging from simplest to advanced approaches. The multifaceted spatial organization of cell–cell, cell-matrix, and cell-soluble factor interactions resembling the *in vivo* bone microenvironment has been recreated in a three-dimensional (3D) manner.^6–8^ While a wide range of bioengineered strategies with suitable biophysical and biochemical cues have been proposed, approaches that exploit the ability of the cells to self-organize into constructs with a highly regenerative capacity have become a top priority in the design of strategies toward bone regeneration.^9^ Despite encouraging findings, the utilization of the bioengineered bone microenvironment is still poorly translated to human clinical trials, in part due to our limited ability to understand, modulate, and control the regenerative steps, the re-vascularization process, and the host's response that usually results in failure of the BTE strategy.^10–12^

In this Perspective, bioengineering bone microenvironments recapitulating key characteristics of the bone regeneration pathway hold great promise to provide new treatment concepts for replacement, regeneration, and healing of bone. To this end, miniaturized bone platforms can assist in (i) investigating cellular and noncellular components of the native bone microenvironment as well as their highly orchestrated interplay; (ii) combining different biomaterials, properties, and technologies to design approaches with high biological complexity and regenerative capacity; (iii) synchronizing cells with biochemical and physical cues in a regulated manner able to guide and improve new bone tissue formation; (iv) exploiting biocompatible and immunomodulatory strategies to avoid immune rejection of the implant; (v) identifying the appropriate strategy for personalized therapy; and (vi) achieving reproducible performance parameters required to facilitate clinical translation. Alternatively, the development of such technologies can also be extremely useful for the *in vitro* basic research in bone biology, assessment of novel pharmaceutical formulations for bone/bone marrow pathologies, and as an alternative to reduce animal experimentation.

## II. THE HUMAN BONE MICROENVIRONMENT

**FIG. 1.**
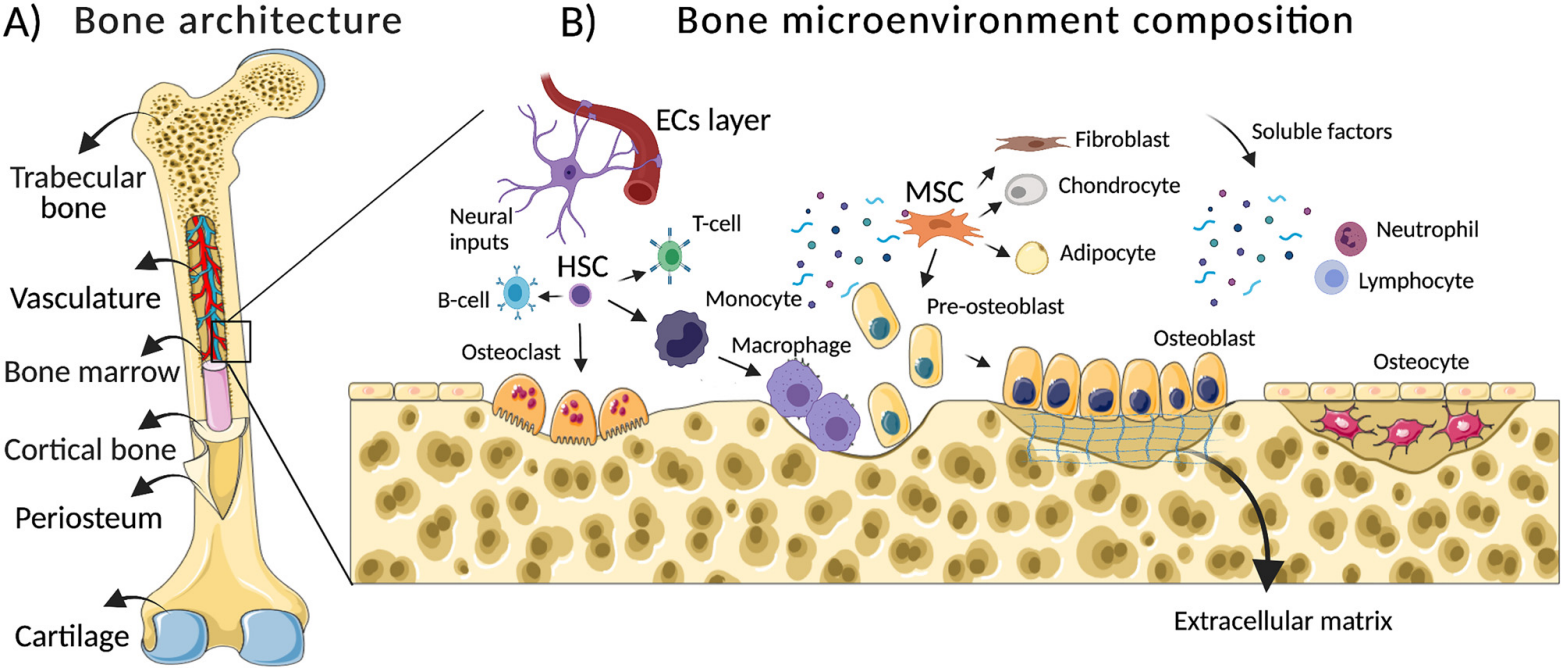
Simplified representation of the bone microenvironment: from architecture to composition. (a) Long bones have a distinct hierarchical tissue organization. Histologically, bone is subdivided into (1) trabecular bone, (2) bone marrow, (3) cortical bone, (4) periosteum, and (5) cartilage. Differences in their composition, density, and porosity give the bone distinct mechanical and regenerative properties. (b) The bone microenvironment has a dynamic composition characterized by an orchestrated interaction between cellular components (skeletal cells, stem cells, vascular cells, immune cells, and nerve cells) and noncellular components (e.g., extracellular matrix, soluble signals, and vascular networks). ECs (endothelial cells), HSC (hematopoietic stem cells), and MSC (mesenchymal stem cells).

Histologically, long bones are subdivided in the following osseous tissues: (i) bone marrow (the soft tissue that fills the central bone intra-medullary channel where hematopoiesis takes place), (ii) trabecular bone (a tissue with an irregular structure and high porosity, 50–90 vol. %), (iii) cortical bone (the hard-outer layer with an ordered structure and low porosity-10 vol. %), (iv) periosteum (a connective tissue along the outer surface of bone comprised by osteoprogenitor cells, which plays an indispensable role in bone healing), and (v) cartilage (a flexible tissue that covers the ends of bones to create a low-friction environment and cushion at the joint) [Fig. 1(a)].

This specialized 3D microenvironment is characterized by synchronized secretion of signaling molecules by skeletal cells, namely, osteoblasts, osteoclasts, and osteocytes, which are jointly responsible for the dynamic regulation of the bone tissue remodeling.^13^ Basically, these skeletal cells support bone remodeling by three distinct processes: (1) osteoclastogenesis, which consists in the removal of old or damage bone tissue by osteoclasts, (2) osteogenesis, the subsequent new bone formation by osteoblasts, and (3) the regulation of osteoclast/osteoblast activities by osteocytes to maintain the balance between bone resorption and bone formation.^14^

Despite skeletal cells, the bone microenvironment is also rich into multipotent stem cells, namely, mesenchymal stem cells (MSCs) and hematopoietic stem cells (HSCs)^15^ [Fig. 1(b)]. While MSCs are known for their capacity to differentiate into osteoblasts, adipocytes, chondrocytes, and fibroblasts,^16^ HSCs give rise to lymphoid and myeloid lineage cells, including osteoclasts in a process called hematopoiesis.^17^

Remarkably, endothelial cells (ECs) are the most common mature cells found in the bone microenvironment.^18^ In a close cellular crosstalk between skeletal, stem cells, and ECs, these are the key cells that are responsible for maintaining bone homeostasis, regeneration, and restoration in the case of bone damage.^19^ Furthermore, recent data have shown that immune cells, namely, monocytes, macrophages, neutrophils, dendritic cells, natural killer cells, and B and T lymphocyte subsets, closely interact with skeletal cells in a so-called osteo-immune microenvironment.^20,21^ Despite the direct role of immune cells in the response to infection, inflammation, and tumor initiation or progression, accumulating evidence has demonstrated that these cells also play a critical role in the regulation of osteogenesis and osteoclastogenesis.^22^

In addition to cellular components, bone microenvironment is also enriched by several noncellular elements, namely, extracellular matrix (ECM), soluble factors, and vascular networks. The ECM components consist of inorganic (±60%) and organic elements (±40%).^23^ While the inorganic phase of bone is composed of minerals, i.e., calcium and phosphate in the form of hydroxyapatite (HA) crystals, the organic part is comprised of collagen type I (±90%) and noncollagen proteins (±10%).^23,24^ In general, the ECM components secreted by cells into the bone microenvironment at the extracellular space, most notably HA crystals, collagen type I, III, and IV, fibronectin, laminin, osteopontin, osteonectin, and osteocalcin, provide tissue mineralization, integrity, elasticity as well as stem cell regulation and bone turnover.^23–25^ The transmission of biochemical and mechanical stimuli by HA cues, namely, the effect of HA crystals on elastic modulus/hardness/deformity and the release of calcium and phosphate ions affect the behavior of stem cells, plays a critical role in the regulation of osteogenesis.^24^

Furthermore, several soluble molecules, i.e., cytokines, chemokines, and growth factors, have crucial roles in the cell signaling activity within bone inter-space.^20,26^

All these components that include a multiphenotypic cellular phase, an inorganic/organic (ECM) phase, soluble factor phase, and the vascular network provide a highly dynamic bone microenvironment [Fig. 1(b)]. Although its exact composition differs based on sex, age, and health conditions, orchestrated interactions of the components are essential for the maintenance of bone architecture (mass/functionality) and during repair.^24^ Consequently, dysregulation in such composition and interactions have been correlated with metabolic bone disorders (e.g., osteoporosis and osteopetrosis), tumor initiation/progression, and a decrease in the regenerative capacity in the case of severe bone fractures.^27^

Currently, the bone microenvironment (i.e., its structure, components, and interactions) has been extensively explored as a potential therapeutic tool to control, modulate, and improve the bone's regenerative capacity. For that, a deeper understanding of the bone microenvironment during the repair process has facilitated the identification of key players involved in such a coordinated mechanism.

For example, it is known that after a bone fracture, a sequence of events occurs at the injured tissue, namely, skeletal integrity loss, bone vasculature disruption, hematoma formation, and inflammation.^4^ Locally, the bone regenerative mechanisms are activated in a set of finely coordinate processes. Succinctly, these mechanisms are divided into three main stages: (1) resolution of inflammation, (2) bone healing, and (3) bone remodeling.^28^

For the resolution of inflammation, immune cells (monocytes, macrophages, neutrophils, granulocytes, and lymphocytes) play the most important role at this stage.^1^ Cytokines with synergistic or antagonistic effects are secreted in a spatiotemporal manner to overcome the inflammatory phase. At the same time, immune cells also (i) recruit ECs to participate in the formation of new blood vessels, (ii) recruit and promote fibroblast proliferation to induce fibrosis of the fibrin clot, and (iii) recruit MSCs regulating their proliferation and osteogenic differentiation.^28^ Gradually, macrophages transform from M1-type polarization to M2, secreting anti-inflammatory cytokines and modulating the local bone microenvironment in favor of a pro-regenerative state.^21^

In the next stage, MSCs are stimulated by cytokines and hypoxic conditions to proliferate and differentiate into osteogenic cells. In this phase, the bone healing mechanisms may occur via an intramembranous or endochondral pathway, depending on the bone anatomical position, size, and vascular supply. While intramembranous ossification occurs directly by the differentiation of MSCs into osteoblasts, endochondral ossification is an indirect mechanism mediated via MSCs differentiation into chondrocytes and subsequent replacement of the cartilaginous template by new bone tissue.^29,30^ Herein, along the mesenchymal differentiation process into osteoblasts or chondrocytes, nonmineralized and mineralized matrices are secreted by the cells. In the last phase of bone healing, the bone is restored to its original shape and mechanical properties by the bone remodeling mechanism. This stage is supported by the crosstalk between osteoblasts and osteoclasts to provide the appropriate architecture and functionality to the new bone by orchestrating relative bone formation and resorption activities.

In summary, the current knowledge about the bone microenvironment and its native reparative properties has been used to artificially simulate how its components orchestrate the regenerative mechanisms.

## III. BIOENGINEERED BONE MICROENVIRONMENT-BASED STRATEGIES: FROM SIMPLEST TO HIGHLY COMPLEX APPROACHES

**FIG. 2.**
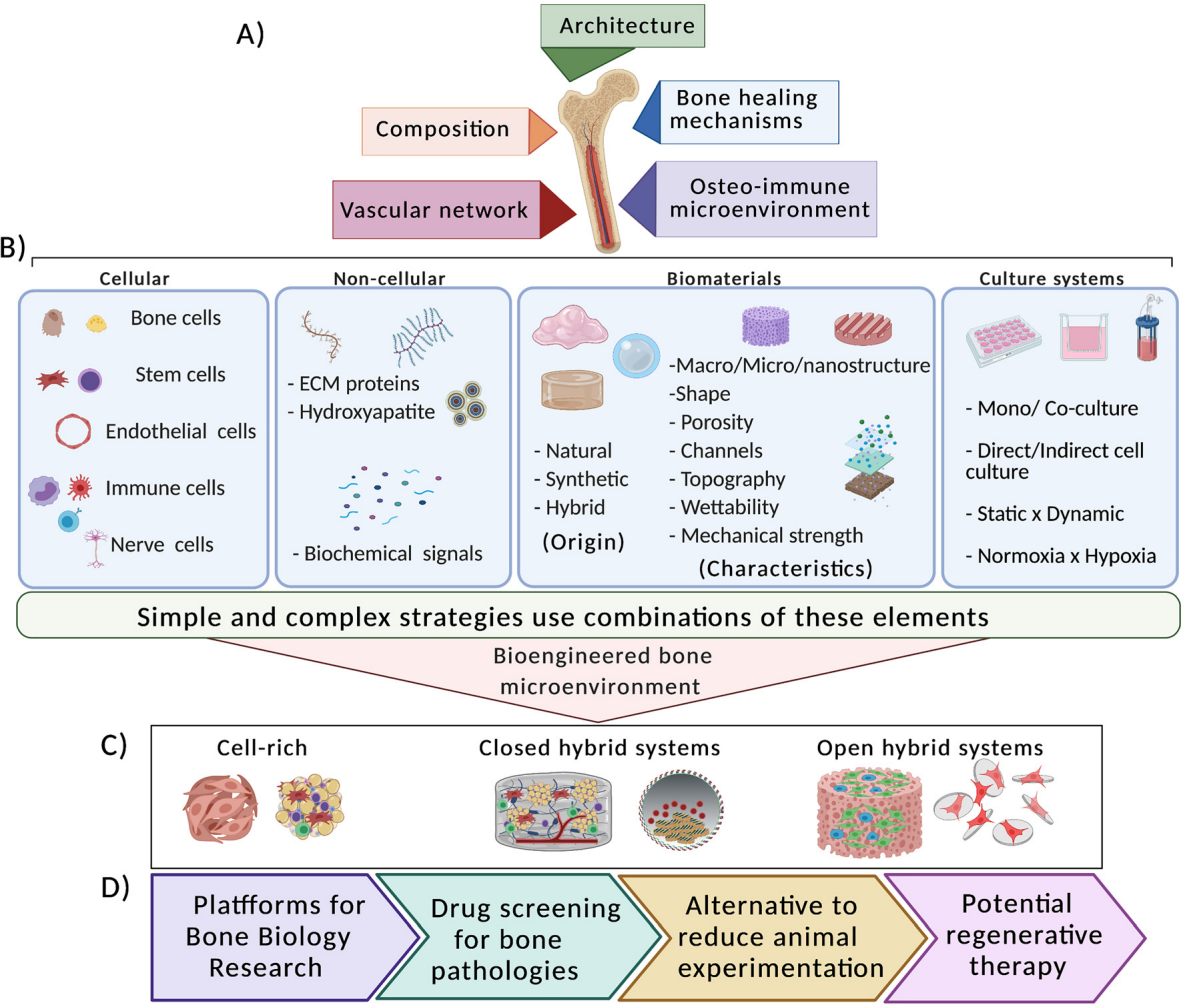
Schematic representation of the most common components used to recapitulate the bone microenvironments under *in vitro* conditions and its potential applications. (a) Simple and complex strategies have been proposed to mimic the bone architecture, the composition, the bone healing mechanisms, the vascular network, and the osteo-immune microenvironment. (b) The strategies combine different elements, i.e., cellular and noncellular components, biomaterials, technologies, and culture systems. (c) The development of bioengineered bone microenvironments can be divided into cell-rich (e.g., scaffold-free and cell-sheets), closed hybrid systems (e.g., scaffolds, hydrogels, and liquefied capsules), and open hybrid systems (e.g., porous scaffolds, particles, fibers, or membranes). (d) Three-dimensional (3D) constructs resembling the *in vivo* bone complexity can find a wide range of applications. They can be used as platforms for basic biology research, drug screening for bone pathologies, as alternative to minimize animal experimentation, and as potential regenerative therapy.

The challenge of the reconstructive surgery to repair large bone fracture and defects has inspired the development of numerous *in vitro* constructs designed to stimulate regeneration and healing *in viv*o.^31,32^ To give a practical contribution to this issue, a deeper knowledge of the bone microenvironment can provide tools to modulate and control the bone regenerative capacity. To this end, several strategies based on the understanding of the bone microenvironment have been proposed.^8,33–35^ Taking into consideration the bone tissue complexity, we highlight strategies that recapitulate specific bone properties, namely, its (i) architecture, (ii) composition, (iii) bone healing mechanisms, (iv) bone vasculature network, and (v) the osteo-immune microenvironment [Fig. 2(a)].

From simplest to complex, strategies usually combine numerous components of the well-known tissue engineering (TE) triad (cells, bioactive signals, and biomaterials) [Fig. 2(b)]. The concept of “simple” is correlated with the capacity of single triad components guide the regenerative bioperformance at the injury site. For example, biomaterials with tunable biophysical and biochemical characteristics have been fabricated to facilitate the attachment and migration of host stem and progenitor cells, drive the differentiation of these cells into bone tissue-specific cell types, and/or modulate the local immune response in a favor to a pro-regenerative state.^9,12^ In contrast, complex strategies, also known as “hybrid systems,” combine several components, i.e., cellular/noncellular elements, biomaterials, technologies, and different culture systems, to recreate biologically relevant constructs with ability to recapitulate the native bone microenvironment as close as possible.^32,36,37^

These BTE approaches provide the advantage of investigating diverse components of the bone microenvironment as well as their synergic interactions, trying to clarify a cascade of coordinated events in which the bone regeneration process involves. In this sense, a wide range of bioengineered bone microenvironments have been developed to provide appropriate cell adhesion sites and positively influence cells to self-organize into hierarchical structures. Briefly, 3D bone constructs are further divided into cell-rich (e.g., scaffold-free and cell-sheets), closed hybrid systems, and open hybrid systems [Fig. 2(c)]. While closed systems encapsulate cells in 3D structures which do not allow direct cellular contact with the external environment (e.g., cells seeded in scaffolds, hydrogels, and liquefied capsules), open systems allow a direct cellular contact with the external environment (e.g., cells seeded at the surface of porous scaffolds, particles, fibers, or membranes). The bioperformance of such bioengineered bone microenvironments will be discussed in detail along the text.

Overall, the development of such strategies has been useful not only to understand straightforward basic aspects of the native bone tissue, but also for the design of innovative strategies with high biological complexity. Of note, the fabrication of miniaturized bone platforms may also find a broad of applications, ranging from basic platforms for bone biology investigation to potential clinical use [Fig. 2(d)].

### A. Mimicking the bone architecture

Bone architecture and function are maintained by a homeostatic load-adaptive remodeling. The constant bone remodeling is influenced by genetic, environmental factors, age, and bone pathological conditions.^36^ In addition, mechanical loading generated by physical activity or diminished by inactivity/obesity also exert potent effects on bone homeostasis. These factors influence the continuous bone formation/resorption mechanisms and determine whether the bone remodeling promotes gain or loss of bone mass.^25^

The different bone tissues are anatomically arranged to accommodate stress demands (i.e., force/area) and strain demands (i.e., original shape deformation) applied to the skeleton under different stimulus.^5^ In fact, the multiscale bone architecture and the mechanical loading have inspired researchers to design microenvironments, which capture essential characteristics of the bone tissue microenvironment from its macro- to nanostructure. While simple strategies have recapitulated basic aspects of the bone tissues, complex approaches have incorporated hierarchical structures, namely, Haversian channels, Volkmann-like channels, and osteon-mimetic structures to mimic the native architecture of the bone as closely as possible. To this end, 3D and 4D bioprinting technologies have become attractive bottom-up alternatives to scaffolds prepared via conventional top-down methods.^38^ Such technologies have recreated the bone microenvironment with customized structures, namely, shape, macro/micro/nanostructure, porosity, channels, wettability, and mechanical strength with ability to control and/or modulate bone resident cells behavior.^32,38,39^

Of note, biomimetic strategies have attracted much attention to construct osseous architectures with superior performance for bone replacement. While the regenerative properties of the cortical bone are quite limited, special attention should be given to the trabecular bone and periosteum due to their superior remodeling capacity. Taking into consideration that 80% of the bone remodeling processes occur in the trabecular bone, biomimetic strategies that effectively recapitulate such mechanism should be a priority during the design of bone-regenerative approaches. For example, a bone–mimicking bioceramic scaffold fabricated by digital laser processing (DLP)-based 3D bioprinting technology was seeded with MSCs into the trabecular bone portion of the scaffold.^32^ In contrast, ECs and Schwann cells were seeded on Haversian channels, under controlled compressive strength and porosity. The biomimetic recapitulation of the trabecular bone microstructure combined with key bone resident cells showed a significant improvement of osteogenic, angiogenic, and neurogenic properties. Interestingly, after implantation in femoral bone defects of rabbits, a considerable amount of new bone formation and blood vessel ingrowth were observed in the coculture scaffold compared to the monoculture scaffold and the cell-free scaffold. These results emphasize the ability of the transplanted coculture cells to self-organize and orchestrate a bone formation mechanism in a bone defect. However, there is no information about osteoclastogenesis activity or how this could influence the bone formation process.

The design of bone-specific personalized shapes and structures should provide not only biomimetic physical factors for the transplanted cells, but also a favorable biomechanical environment for both transplanted and native cells to promote an efficient bone remodeling. Instead of excessive attempts to recapitulate the anatomical architecture of bone at all different length scales in tissue engineering constructs, future strategies should strive to provide the right signals for the multicellular colonization orchestrate the well-balanced actions of bone remodeling with ability to coordinate bone formation/resorption, a physiological mechanism that generates sophisticated bone microarchitecture and effectively restore bone function.

### B. Mimicking the bone microenvironment composition

Substantial efforts have been made to mimic the native bone composition to exploit the ability of the cellular and noncellular components to self-organize and interact with each other. To this end, researchers have taken inspiration from (i) the capability of stem cells [e.g., MSCs and adipose-derived stem cells (ASCs)] to differentiate into osteogenic lineage cells,^8,30,40^ (ii) the crosstalk between osteoblasts and osteoclasts to understand their key role during the remodeling process,^35,41^ (iii) the capacity of cytokines and/or growth factors as biochemical signals to stimulate bone regeneration,^42^ and (iv) the importance of the ECM components for bone mineralization during the process of new bone formation and healing.^23,24^

#### 1. Biomimetic environment: Bone cellular composition

To mimic the cellular composition of the bone microenvironment, strategies have used a combination of mono-/coculture seeded in different matrices and maintained in (1) indirect co-culture or (2) direct culture under static conditions.^41,43,44^ Herein, the focus is to exploit and understand basic mechanisms that control and improve cell adhesion, proliferation, differentiation, ECM deposition, and the paracrine cell signaling during bone formation and repair. While most of the biomimetic strategies maintained under static condition reveal a plethora of components and properties found in the native bone microenvironment, they fail to recapitulate the dynamic nature of the bone microenvironment. The depletion of nutrients and oxygen, the incomplete removal of waste products at the interior of the matrices, and the formation of a necrotic core at prolonged culture have been reported as main disadvantages of using static systems.^45^

To solve these issues, the combination of conventional matrices in dynamic systems promotes (i) the circulation of nutrients and oxygen, (ii) the removal of waste products, and (iii) a better biophysical stimulus for cell-driven differentiation. Compared to static systems, dynamic approaches provide 3D constructs with higher biological complexity,^46^ a promising strategy to recapitulate more accurately the bone microenvironment composition under *in vitro* conditions. In this sense, a variety of bioencapsulation strategies under dynamic systems have been designed.^7,8,42^ For example, ASCs and osteoblasts were co-encapsulated with surface functionalized microparticles in liquefied microcapsules cultured in spinner flasks.^8^ While the liquefied environment allowed cells to self-organize, the dynamic environment successfully promoted an *in vitro* bone-like tissue formation with a mineralized and more organized ECM deposition compared to the static system. The continuous diffusion of essential nutrients emphasizes the efficiency of the dynamic system to support cell proliferation, cell-intercommunication, and cell-driven differentiation. We anticipate that this tunable platform might be an exciting strategy to recapitulate the heterogeneity of the bone microenvironment and to exploit key components responsible for bone formation, regeneration, and healing. To this end, increasing the cellular complexity and/or the combination with multiple signaling cues or bioinstructive biomaterials in a regulated manner could recapitulate a multifaceted bone-related microenvironment under *in vitro* conditions.

Regarding the cellular diversity of the bone microenvironment, tri-coculture approaches in dynamic systems have emerged as an advanced strategy to bioengineer part of the multiphenotypic microenvironment. For example, osteoblasts/osteoclasts/endothelial cells seeded in the porous ceramic scaffold in a perfusion-based bioreactor were able not only to mimic the ECM remodeling components but also showed characteristics of an accelerated *in vitro* bone formation, showing the presence of bone sialoprotein, matrix deposition (by C-terminus procollagen-I propeptides), resorption (by N-terminus collagen-I telopeptides and phosphate levels), and osteoclastic activity (by TRAP-5b).^35^ After subcutaneous implantation in rodents, the engineered bone-microenvironment promoted a humanized bone-like tissue formation with localized blood vessels. Although promising results were obtained from this approach, this and similar works did not report in detail key fluid flow parameters inside the matrices, which may influence the shear stress, an essential characteristic for cell behavior in terms of cell attachment, migration, proliferation, and differentiation.^8,32^ However, most of tri-coculture approaches in dynamic systems should also exploit the viability of the transplanted cells, the immune response around the implant as well as the rate of the scaffold/hydrogel degradation over time.

Despite the emergence of successful approaches to recreate the bone microenvironment based on its composition, numerous strategies do not capture the synchronized activity of the cellular and noncellular components as well as the intricate cell signaling (autocrine and paracrine interactions) during bone tissue formation, regeneration, and healing. In addition, when translated to *in vivo*, most of the 3D constructs are implanted in ectopic sites, which significantly differ from the osseous microenvironment of orthotopic applications.

#### 2. Biomimetic environment: Bone ECM components

Despite its multicellular composition, the bone microenvironment is also rich into extracellular matrix, i.e., inorganic and organic components. While the inorganic components consist of a mineral part consisting of hydroxyapatite (HA) crystals, the organic components mainly consist of collagen and noncollagenous proteins.^28^ Inspired by the importance of the bone microenvironment structure and functionality,^23,24^ strategies have proposed ECM-biomimetic environments to optimize the osteogenic, chondrogenic, and angiogenic potentials of the ECM components.^47,48^

From synthetic or natural materials, HA, collagen, and fibronectin have been commonly used for the preparation of ECM-based scaffolds/hydrogels.^15,23,49,50^ For example, a bioinspired scaffold containing HA-silica core–shell nanorods seeded with MSCs and ECs promoted osteogenic differentiation *in vitro.*^51^ When translated to *in vivo* conditions, studies in the chicken chorioallantoic membrane demonstrated good biocompatibility and vascularization potential. Beyond that, after implantation in femoral rat defects, this nanocomposite scaffold was able to induce new bone formation accompanied by the degradation of the material. Likewise, collagen scaffolds seeded with human dental pulp stem cells (DPSCs) and implanted in critical-size rat calvarial defects significantly promoted new bone formation compared to untreated defects.^48^ Interestingly, the bone defect repair was correlated with the expression of osteogenic alkaline phosphatase (ALP) and secretion of type I collagen, indicative of osteogenic differentiation and tissue mineralization. Although ECM biomimetic environments have demonstrated biocompatible/biodegradable properties and the ability to promote osteogenic differentiation, both *in vitro* and *in vivo*, the effect of the ECM components in the bone remodeling process should also be considered, namely, for orthotopic applications.

In this sense, ECM-based biomimetic strategies have been designed to clarify the role of the ECM, not only for bone formation but also for bone remodeling. For example, a direct co-culture of osteoblasts and osteoclasts in a hydroxyapatite scaffold cultured under a static system mimicked the complex bone-building and -resorbing activities of these cells, similar to their *in vivo* state.^41^ When translated to *in vivo* conditions, this bioengineered strategy promoted mature bone-like tissue formation after subcutaneous implantation in rodents. These findings emphasize that ECM components have the ability to promote bone formation and remodeling by regulating the bone resident cells behavior through biochemical, biophysical, and mechanical cues.^24^

Although ECM-biomimetic scaffolds based on different ECM components can improve bone defect repair, they fail to recapitulate the native ECM diversity, its intricate network, and interactions. To overcome these limitations, decellularized ECM scaffolds have the advantage of maintaining the native ECM components, providing the original microarchitecture, bioactivity, and the flexibility of the bone microenvironment.^24,52^ This strategy gives mechanical support for bone resident cells and directly affect migration, elongation, proliferation, and cell fate.^47^ Decellularized ECM scaffolds facilitate the recruitment and differentiation of host cells with instructive niches for tissue regeneration.^49^ Nevertheless, a deeper understanding of how the combination of inorganic and organic ECM components effectively provides a favorable environment for osteogenesis, chondrogenesis, angiogenesis, and inflammatory modulation should be obtained to clarify the biological performance of such novel technologies. Currently, this knowledge has become crucial for the development of advanced biomedical applications with high similarity to the structural and functional properties of the native bone microenvironment.

### C. Mimicking the native bone healing mechanisms

Based on the native bone healing mechanisms, several approaches have explored the potential of MSCs to differentiate into osteoblast or chondrocytes as template for new bone formation. Herein, miniaturized bone microenvironment constructs not only should promote the stem cell proliferation and differentiation potential, but also support the development of an osseous tissue with appropriate vascularization and immune response upon implantation. However, the combination of these regenerative properties in a single approach remains a challenge.

Stem cells, particularly MSCs, have been intensively explored in the field of BTE strategies due to their self-renewal, multipotentiality, anti-inflammatory, immunomodulatory, and pro-angiogenic effects.^16,44^ To this end, several signals including biochemical, mechanical, geometrical, and topographical have been used in combination with MSCs to recapitulate the ossification mechanisms. Traditionally, simple strategies combine MSCs seeded in conventional matrices with biochemical factors (e.g., bone morphogenetic proteins) or small molecular compounds (e.g., dexamethasone, β-glycerophosphate, and ascorbic acid) to induce osteogenic or chondrogenic differentiation.^30^ However, the use of soluble factors involves some limitations and risks, namely, their short-term bioactivity both *in vitro* and *in vivo*. Taking into consideration the importance of the biochemical factors required for bone formation and healing, biomaterials with the ability to provide a localized and controlled release of the bioactive molecules involved in such processes have been investigated.^53,54^ Interestingly, strategies able to recapitulate and control its intrinsic signaling on a spatiotemporal manner could provide a positive influence for bone tissue regeneration after implantation.

Alternatively, the use of advanced biomaterials with tunable features has emerged as a strategy to modulate the differentiation of MSCs and the subsequent bone formation in a more efficient manner.^55^ In turn, biomaterials can be used not only as structural support for MSCs, but also as bioinstructive templates for osteogenic and chondrogenic differentiation depending on its composition, geometrical shape, surface topography, and matrix stiffness. For example, surface topography at the nanoscale (i.e., roughness, patterns, and porosity) can promote significant changes in the cytoskeletal organization, cell shape, migration, proliferation, and osteogenic differentiation.^56^ Despite the modulation of MSCs behavior in a controllable manner, these technologies should take in consideration not only the osteogenic potential of MSCs but also their secretome, a key repertoire of bioactive molecules with angiogenic and immunomodulatory properties. The MSCs secretome plays an intrinsic cellular communication in the native bone microenvironment, which is responsible for the recruitment, activation, proliferation, and differentiation of bone resident cells, both in homeostasis and during the bone regenerative process.^16^ Herein, the use of advanced biomaterials should also investigate the MSCs secretome as a potential cell-free therapeutic strategy for bone formation and repair.

Sophisticated strategies with the ability to recapitulate an *in vivo* bone formation under controlled physical and chemical parameters have been proposed. For example, microfluidic chip platforms have shown advantages over other 3D cell culture approaches due to the improved control of physiologic flow velocities, shear stresses, or oxygen gradients. Of note, on-chip systems such as osteogenesis-on-a-chip,^37^ bone perivascular-niche-on-a-chip,^6^ and bone-marrow-on-a-chip^57^ have been reported. For example, a photocurable scaffold embedded within a silicone-based polymer seeded with MSCs was placed into a microfluidic chip for up to three weeks under physiologic flow conditions.^37^ The bioengineered bone microenvironment successfully recreated a favorable environment for osteogenic differentiation of cells in comparison to the same culture under static flow. Although these platforms support a more complex organization in a controlled environment, many microfluidic bone-microenvironment-on-chips do not consider the shear stress exerted by blood flow, stromal, and immune cells. Furthermore, the optimization of several parameters that influence cell fate, including the architecture, nutrient exchange, physical, chemical, and electrical stimulation, could minimize the experimental variability and maximize the performance reproducibility. Despite its operational complexity, the ability of this strategy to synchronize multiple cells with biochemical and physical cues in a regulated manner can provide unprecedent insights into the ossification mechanisms, both in health and in pathological conditions.

### D. Mimicking the bone vasculature network

With a typically stratified organization, the bone vasculature is an intricate network of endothelial cells (ECs) layers regulated by numerous bone resident cells, namely, osteoblasts, MSCs, HSCs, perivascular cells, and immune and nerve cells.^58^ The vasculature is of fundamental importance for bone homeostasis and during fracture repair due to its delivery of oxygen and nutrients, removal of waste products, and its key role in the recruitment and regulation of reparative cells to the injured site.^59^ In the case of large bone fractures/defects, the vascular network is usually disrupted that creates a local hypoxic environment and complicates or even impairs the natural process of bone tissue regeneration and healing.^60^ This highlights the imperative role of vascularization during the various steps in bone healing. Consequently, the development of 3D approaches with re-vascularization potential is still a top priority for BTE strategies. To develop vascular microstructures resembling the *in vivo* microenvironment, extensive investigation has been dedicated to this field during the past decades.

**FIG. 3.**
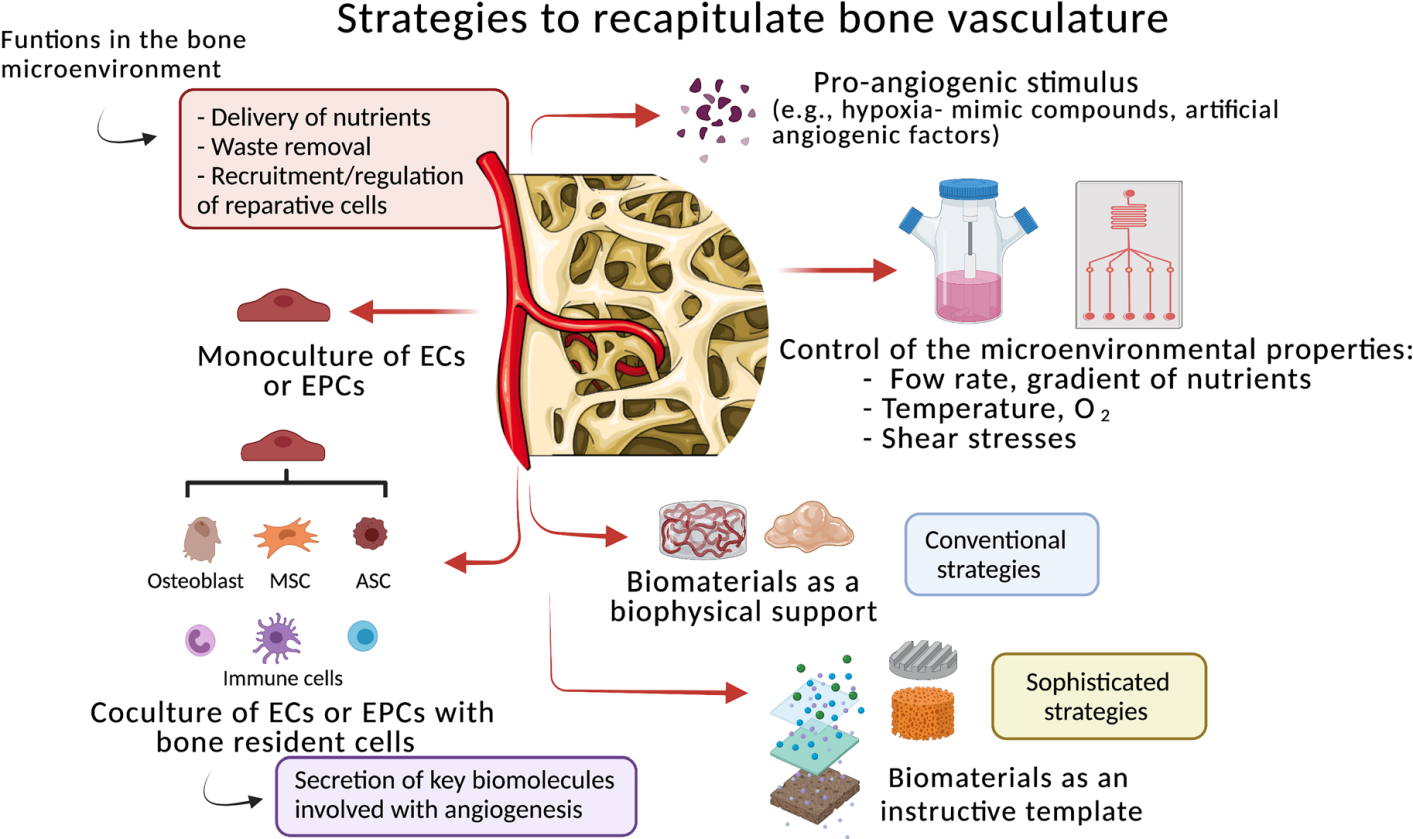
Strategies to mimic the bone vascular networks. The strategies use monoculture of endothelial cells (ECs) or endothelial progenitor cells (EPCs), or coculture of ECs/EPCs with bone resident cells. Different approaches use numerous biomaterials as a biophysical support or as a bioinstructive template. The strategies can also combine different technologies to recapitulate the bone microenvironmental properties [e.g., physiological flow, gradient of nutrients, temperature, oxygen (O_2_) tension, and shear stresses], under controlled *in vitro* conditions. The supplementation of pro-angiogenic factors is also a common practice. MSC (mesenchymal stem cell) and ACS (adipose-derived stem cell).

Conventional strategies combine monoculture of ECs or coculture of ECs with bone resident cells seeded in traditional scaffolds/hydrogels, in the presence or absence of pro-angiogenic factors (Fig. 3). Herein, coculture systems seem to be more appropriate approaches compared to the monoculture, due the secretion of essential biomolecules by the bone supportive cells during the development and stabilization of the microvessels.^33^ However, despite the establishment of essential adhesion sites, cell–cell, and cell–ECM interactions, most of these approaches do not precisely give the support to the encapsulated cells to form and maintain a vascular network inside the 3D construct, both *in vitro* and *in vivo*.

To overcome these shortcomings, sophisticated strategies, namely, the use of biomaterials with micro/nanostructures, different porosity, matrix stiffness, topography, tubular structures, and controlled release of specific biological factors, have received special attention due to their critical influence on ECs behavior and angiogenic potential.^54,61^ For example, a coculture of human umbilical endothelial cells (HUVECs) and MSCs was seeded in a 3D printed biodegradable polycaprolactone (PCL) scaffold with controlled released of deferoxamine (DFO), a well-known hypoxia-mimic compound that stimulates angiogenesis, both *in vitro* and *in vivo.*^62^ The recapitulation of the hypoxic bone microenvironment by this biofunctional scaffold induced abundant formation of vascular patterns accompanied by deposition of mineralized matrix and osteogenic differentiation. After implantation in a rat femur defect, the engineered construct promoted a rapid vascular invasion with subsequent osteointegration. These results emphasize that advanced biomaterials can provide localized angiogenic stimulation in a spatially and temporally controlled manner, which surpasses the exogenous administration of soluble pro-angiogenic factors. However, key parameters correlated with the integration of the pre-vasculature network to the host as well as its stability and functionality over time need to be addressed. In addition, biomaterials with controlled features (e.g., topographical/geometrical pattern and pores) significantly influence the endothelial orientation, proliferation, and migration during the formation of a microvascular network under *in vitro* conditions.^63^ Interestingly, this suggests that the combination of biochemical and biophysical cues with coculture of ECs and bone resident cells contribute to achieve a better formation, maturation, and stabilization of the microvessels under *in vitro* conditions.

Cell sheet (CS) technology has emerged as an alternative to the use of scaffolds.^64^ This powerful approach can recreate a vascularized bone microenvironment without the need of a scaffold as a support. For example, a stratified and hierarchical 3D vascularized network was developed by magnetically labeling HUVECs and ASCs in a triple CS conformation with HUVECs between two sheets of ASCs.^40^ The presence of specific osteogenic and angiogenic factors successfully showed that this platform supports cell adhesion, proliferation, and cell-intercommunication able to mediate deposition of mineralized matrix and formation of a stratified bone microtissue under *in vitro* conditions. Furthermore, its implantation in chick chorioallantoic membrane (CAM) showed not only an organized cell capillary-like structure but also the integration of human cells in the chick vasculature. These results demonstrate the potential of this kind of technology to create easily multicellular, robust, and reproducible constructs for BTE purposes, but limitations concerning the technology's performance into recapitulating the diversity of the bone microenvironment need to be addressed. Moreover, it is still unknown if such engineered construct promotes *in vivo* vascularized microtissue formation or if it could be necessary to incorporate biochemical/biophysical cues provided by biomaterials, necessary for the formation of a functional bone microvasculature.

Advanced strategies, for example microfluidic technologies, have been extensively explored as a powerful tool to mimic both vasculogenesis and angiogenesis under *in vitro* conditions.^65,66^ These platforms use different matrices in specific areas of the chip with two channels that allow ECs or endothelial progenitor cells (EPCs) to form a microvasculature. Compared to standard 3D approaches, this technology provides a more accurate recapitulation of the native bone vasculature. For example, a functional human tri-coculture (ECs, bone marrow-derived MSCs, and breast cancer cells) within a 3D native bone matrix successfully recapitulated a humanized bone perivascular niche-on-a-chip.^6^ Under controlled fluidic conditions, it was possible to establish a long-lasting, self-assembled vascular network without angiogenic supplementation. The MSCs assumed phenotypical transition toward perivascular cells, which supported capillary-like structures lining the vascular lumen. However, it is not clear if angiogenesis was optimally promoted by the controlled conditions or by cell–cell interactions. Of note, despite its difficult handling, the standardization, and scale-up for BTE applications, this type of technology becomes a powerful tool to exploit several parameters involved in the bone regenerative process. For example, the role of pro- and anti-inflammatory stimulation in the formation of vascular network is a field still poorly explored. Additionally, by increasing its complexity by introducing representative bone resident cells, this technology has great potential to be used for drug toxicology tests, with the advantage of reducing animal experimentation.

The recreation of a stable and functional vascular network under *in vitro* conditions with the ability to maintain its post-implantation properties continues to be a challenging arena. It is expected that promising approaches may focus on combining different properties of the biomaterials to provide a favorable re-vascularization environment for both transplanted and bone resident cells. For that, critical parameters for suitable stimulation of microvessel formation, stabilization, and functionalization need to be improved.

### E. Mimicking the osteo-immune microenvironment

Despite the presence of osteolineage cells, the bone microenvironment is the largest reservoir of hematopoietic stem cells (HSCs), lymphoid/myeloid progenitors, and several mature immune cells, i.e., monocytes, macrophages, dendritic cells, natural killer cells, and B and T lymphocytes.^20^ These cells interact with bone and bone marrow cells in a synchronized activity responsible by the regulation of osteogenesis, osteoclastogenesis, infection, inflammation, and anti-tumor response.^67^ In the last few years, the in-depth understanding of the osteo-immune microenvironment, namely, its main cell key players and interactions, has been considered as crucial for the development of innovative regenerative medicine therapies.

In this sense, bioengineered bone microenvironments have tried to focus not only on direct osteogenesis but also to provide a favorable immune response to support the implant integration.^20,68^ Among various immune cells, macrophages have received special attention by their relevant role in the osteo-immune regulation. Briefly, the secretion of cytokines by macrophages recruits other immune cells that initiate a foreign body reaction, inflammatory modulation, and subsequent bone regeneration and healing.^68^

Given such importance, many strategies have recapitulated the modulation of the macrophage polarization in a favor to a pro-regenerative type-2 phenotype (M2) to create an ideal environment for osteogenesis.^21,42,69,70^ Simple approaches have focused on the interaction between biomaterials and cells, namely, (i) immune cells, (ii) bone cells, and (iii) stem cells.^70,71^ Findings have shown that while traditional biomaterials are correlated with a macrophage M1 polarization (pro-inflammatory) and several adverse immunogenic host reactions,^72^ advanced biomaterials provide bioinstructive signals able to modulate the immune response for an anti-inflammatory state.^73^ These results encourage the design of a next generation of BTE strategies, which explore biomaterials to effectively provide an appropriate modulation of the local immune system in response to the implant.

Of note, some strategies have explored the *in vivo* immune response upon 3D construct implantation without any transplanted cells.^74,75^ For example, a subcutaneous implantation in mice of a conventional polycaprolactone (PCL) scaffold and a printed hierarchically structured (“microchanneled”) PCL scaffold showed that while the first one was significantly colonized by activated M1 macrophages, the printed scaffold promoted the recruitment, infiltration, colonization of neutrophils, and M2 macrophages around the implant.^75^ The modulation of the osteo-immune microenvironment around the printed scaffold was also correlated with stem cells homing, and significant release of pro-angiogenic factors when compared to the conventional construct. Such findings demonstrate that the use of microtechnologies can, in general, be useful as a simple strategy to modulate the local *in vivo* bone microenvironment in favor to a pro-regenerative state.

Likewise, micro- and nano-topography have great potential to modulate not only stem cells differentiation and endothelial behavior, as reported above, but also the macrophage behavior and its polarization state.^76^ For example, alumina membranes with a highly ordered nanoporous surface containing pores of different size seeded with macrophages and MSCs showed important adhesive cues for macrophages, affecting their spreading and cell shape.^42^ The nanotopography induced a macrophage modulation to a M2 phenotype with a significant inhibitory effect on osteoclastic activity and increased the release of osteogenic factors, creating an ideal *in vitro* environment for new bone tissue formation and regeneration. Although macrophage modulation is clearly associated with nanotopography-mediated osteogenesis under *in vitro* conditions, it could be also interesting to explore the *in vivo* biological competency of the nanomaterial, alone and in combination with transplanted cells.

Though many biomimetic-osteo-immune approaches have been proposed with interesting findings,^42,50,70,76^ most of them is not yet translated into *in vivo* models. For example, two chemically and texturally different calcium phosphate substrates, namely, a biomimetic calcium deficient hydroxyapatite (CDHA) and a sintered β-tricalcium phosphate (β-TCP), were used to investigate the behavior of macrophages.^50^ The β-TCP significantly reduced the release of pro-inflammatory cytokines compared to biomimetic CDHA. Nonetheless, the microenvironment created after co-culturing MSCs in the macrophages conditioned medium on CDHA showed a potent osteogenic effect. This observation reinforces the idea that osteogenic differentiation may be influenced not only by the nature of biomaterials but also by the local inflammatory environment. However, several exploratory strategies are necessary to understand how different features of biomaterials impact the release of pro- or anti-inflammatory cytokines by macrophages and their osteogenic, osteoclastogenic, and vasculogenic properties.

Considering that not only macrophages but also neutrophils are the first-line cells in contact with the 3D construct upon implantation, the strategies should also intensively explore the neutrophil biology on the context of osteogenesis and clarify its direct/indirect influence on the differentiation of monocytes into macrophages. *In vitro* strategies that diversify the cellular components of the osteo-immune microenvironment to understand the host responses under different stimulus should be considered. For example, the addition of hematopoietic stem cells (HSCs), neutrophils, dendritic cells (DCs), and B and T lymphocytes could be investigated. Taking into consideration that HSCs give rise to both immune cells and osteoclasts,^17^ it becomes necessary to explore the HSC behavior, cell fate, and its therapeutic potential in bone regeneration. These exploratory studies could clarify how HSCs influence the host immune response during homeostasis and the bone regeneration process. In addition to that, neutrophils, DCs, and B and T lymphocytes are also key components of the bone microenvironment, but have been poorly explored in the field of osteo-immune strategies with a focus on bone repair. Herein, liquefied capsules and microfluidic technologies may provide promising exploratory osteo-immune platforms as they are able to compartmentalize nonadherent cells together with adherent cells and biomaterials.^64,66,70^

Overall, the recapitulation of the osteo-immune microenvironment highlights the importance of the crosstalk between immune cells, osteolineage cells, and stem cells during bone repair. However, a deeper understanding of the underlying synergic regulation in such environment is crucial for the design of new immunomodulatory strategies with ability to create an ideal pro-regenerative environment for bone regeneration.

## IV. CONCLUSION AND FUTURE PERSPECTIVES

The design of bioengineered bone microenvironments with high biological complexity and osteo-regenerative properties (e.g., osteogenic, vasculogenic, and immunomodulatory) remains a challenge in the field of tissue engineering. However, the findings herein discussed emphasize that the recreation of an ideal environment for bone formation/resorption and regeneration involves a broad of parameters adequately combined in a coordinated manner.

In fact, while several strategies have demonstrated efficient *in vitro* cellular self-organization and regenerative properties, the capacity of cells to form new bone tissue and the host osteo-integration is usually lost with time, namely, due to poor vascularization and/or a pro-inflammatory immune response around the implant. To overcome these disadvantages, alternatives to improve these two mechanisms before, during, or after the 3D construct implantation should be considered and implemented.

Regarding *in vivo* transplantation of the bioengineered bone microenvironments, the subcutaneous microenvironment does not represent the biological complexity of the bone fracture/defects, which emphasizes the importance of orthotopic instead of ectopic implantation to evaluate bone healing parameters. To this end, standardization, safety evaluation, reproducibility, quality control, and ethical issues are still tasks that need to be overtaken to facilitate the use of orthotopic implants as well as its translation into human clinical trials.

**FIG. 4.**
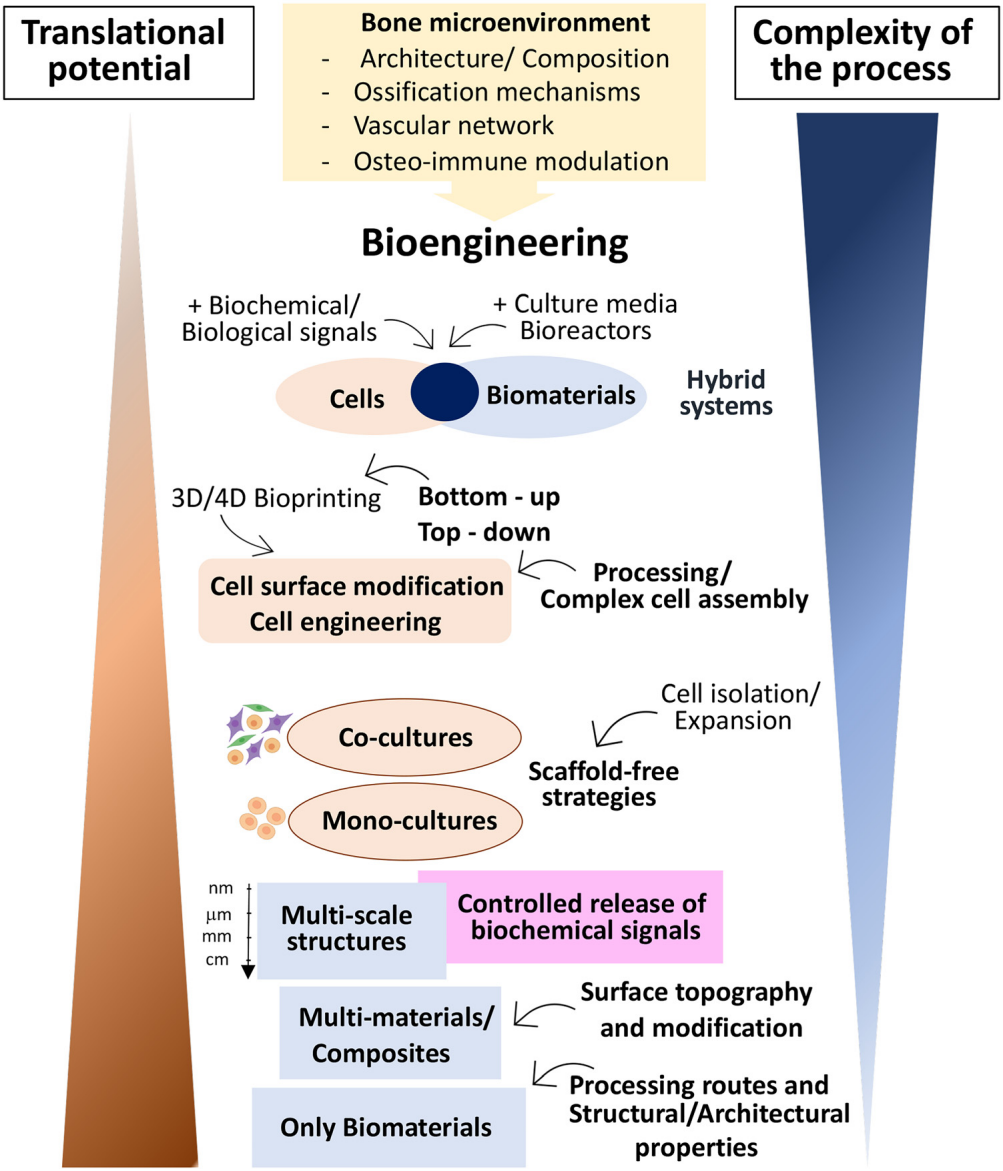
Intrinsic correlation between the process complexity of the bioengineered bone microenvironment and its translational potential. Simple and advanced strategies have been proposed to recapitulate the bone microenvironment. While the first one approach has shown an elevated translation potential, the incorporation of several elements (e.g., biomaterials, cellular and noncellular components, different approaches, technologies, and culture systems) has limited the translational potential of the second approach.

In summary, significant advances have been made over the last few years envisioning the improvement of bone regeneration strategies through the recapitulation of the bone microenvironment. The development of such miniaturized platforms may be an attractive option for bone tissue engineering as they offer small units that able to be implanted with minimal invasive procedure or be assembled in higher scale-devices with controlled architecture using bottom-up procedures. Promising approaches involve biological, biochemical, and biophysical cues combined in a spatial-temporal manner, suitable to orchestrate a self-regulated new bone formation and regeneration. Although encouraging results, the level of complexity of such bioengineered platforms is still inversely correlated with human clinical translation (Fig. 4). To overcome this limitation, significant efforts to achieve translational requirement parameters (e.g., reproducibility, handling, manufacturing difficulties, and long-term *in vivo* response) during the strategy design need to be considered to facilitate the translation of strategies at later R&D stages.

## Data Availability

Data sharing is not applicable to this article as no new data were created or analyzed in this study.

## References

[1] L. Claes , S. Recknagel , and A. Ignatius , Nat. Rev. Rheumatol. 8(3), 133 (2012). 10.1038/nrrheum.2012.1 22293759

[2] M. Orciani , M. Fini , R. Di Primio , and M. Mattioli-Belmonte , Front. Bioeng. Biotechnol. 5, 17 (2017). 10.3389/fbioe.2017.00017 28386538 PMC5362636

[3] L. Li , F. Yu , J. Shi , S. Shen , H. Teng , J. Yang , X. Wang , and Q. Jiang , Sci. Rep. 7(1), 9416 (2017). 10.1038/s41598-017-10060-3 28842703 PMC5572706

[4] D. Lopes , C. Martins-Cruz , M. B. Oliveira , and J. F. Mano , Biomaterials 185, 240 (2018). 10.1016/j.biomaterials.2018.09.028 30261426 PMC6445367

[5] S. L. Manske , C. R. Lorincz , and R. F. Zernicke , Sports Health 1(4), 341 (2009). 10.1177/1941738109338823 23015892 PMC3445123

[6] A. Marturano-Kruik , M. M. Nava , K. Yeager , A. Chramiec , L. Hao , S. Robinson , E. Guo , M. T. Raimondi , and G. Vunjak-Novakovic , Proc. Natl. Acad. Sci. U. S. A. 115(6), 1256 (2018). 10.1073/pnas.1714282115 29363599 PMC5819403

[7] E. Leclerc , B. David , L. Griscom , B. Lepioufle , T. Fujii , P. Layrolle , and C. Legallaisa , Biomaterials 27(4), 586 (2006). 10.1016/j.biomaterials.2005.06.002 16026825

[8] S. Nadine , S. G. Patricio , C. R. Correia , and J. F. Mano , Biofabrication 12(1), 015005 (2019). 10.1088/1758-5090/ab3e16 31443097

[9] C. R. Correia , I. M. Bjorge , S. Nadine , and J. F. Mano , Adv. Healthcare Mater. 10, e2002110 (2021). 10.1002/adhm.202002110 33709572

[10] E. Mariani , G. Lisignoli , R. M. Borzi , and L. Pulsatelli , Int. J. Mol. Sci. 20(3), 636 (2019). 10.3390/ijms20030636 PMC6386828 30717232

[11] E. T. Stace , S. G. Dakin , P. A. Mouthuy , and A. J. Carr , J. Cell Physiol. 231(1), 36 (2016). 10.1002/jcp.25071 26058815

[12] A. K. Gaharwar , I. Singh , and A. Khademhosseini , Nat. Rev. Mater. 5(9), 686 (2020). 10.1038/s41578-020-0209-x

[13] L. J. Brylka and T. Schinke , Front. Immunol. 10, 2182 (2019); 10.3389/fimmu.2019.02182 31572390 PMC6753917

[14] Y. Han , X. You , W. Xing , Z. Zhang , and W. Zou , Bone Res. 6, 16 (2018). 10.1038/s41413-018-0019-6 29844945 PMC5967329

[15] W. Lai , Y. Li , S. Mak , F. Ho , S. Chow , W. Chooi , C. Chow , A. Leung , and B. Chan , J. Tissue Eng. 4, 2041731413508668 (2013). 10.1177/2041731413508668 24555007 PMC3927755

[16] F. Shang , Y. Yu , S. Liu , L. Ming , Y. Zhang , Z. Zhou , J. Zhao , and Y. Jin , Bioact. Mater. 6(3), 666 (2021). 10.1016/j.bioactmat.2020.08.014 33005830 PMC7509590

[17] C. S. Oliveira , M. Carreira , C. R. Correia , and J. F. Mano , Tissue Eng., Part B (published online, 2021). 10.1089/ten.teb.2021.0019

[18] J. Loeffler , G. N. Duda , F. A. Sass , and A. Dienelt , Trends Endocrinol. Metab. 29(2), 99 (2018). 10.1016/j.tem.2017.11.008 29290501

[19] J. Ham , L. Lever , M. Fox , and M. R. Reagan , JBMR Plus 3(10), e10228 (2019). 10.1002/jbm4.10228 31687654 PMC6820578

[20] W. Lin , Q. Li , D. Zhang , X. Zhang , X. Qi , Q. Wang , Y. Chen , C. Liu , H. Li , S. Zhang , Y. Wang , B. Shao , L. Zhang , and Q. Yuan , Bone Res. 9(1), 17 (2021). 10.1038/s41413-021-00141-5 33723232 PMC7960742

[21] M. Maruyama , C. Rhee , T. Utsunomiya , N. Zhang , M. Ueno , Z. Yao , and S. B. Goodman , Front. Endocrinol. 11, 386 (2020). 10.3389/fendo.2020.00386 PMC7325942 32655495

[22] A. Gnoni , O. Brunetti , V. Longo , A. Calabrese , A. L. Argentiero , R. Calbi , G. Solimando Antonio , and A. Licchetta , Oncotarget 11(4), 480 (2020). 10.18632/oncotarget.27439 32064051 PMC6996902

[23] M. S. Carvalho , J. M. S. Cabral , C. L. da Silva , and D. Vashishth , Polymers 13(7), 1095 (2021). 10.3390/polym13071095 33808184 PMC8036283

[24] X. Lin , S. Patil , Y. G. Gao , and A. Qian , Front. Pharmacol. 11, 757 (2020). 10.3389/fphar.2020.00757 32528290 PMC7264100

[25] I. N. Camal Ruggieri , A. M. Cicero , J. P. M. Issa , and S. Feldman , J. Bone Miner. Metab. 39(3), 311 (2021). 10.1007/s00774-020-01168-0 33151416

[26] M. Filippi , G. Born , M. Chaaban , and A. Scherberich , Front. Bioeng. Biotechnol. 8, 474 (2020); 10.3389/fbioe.2020.00474 32509754 PMC7253672

[27] M. B. Greenblatt , J. N. Tsai , and M. N. Wein , Clin. Chem. 63(2), 464 (2017). 10.1373/clinchem.2016.259085 27940448 PMC5549920

[28] G. Zhu , T. Zhang , M. Chen , K. Yao , X. Huang , B. Zhang , Y. Li , J. Liu , Y. Wang , and Z. Zhao , Bioact. Mater. 6(11), 4110 (2021). 10.1016/j.bioactmat.2021.03.043 33997497 PMC8091181

[29] E. J. Sheehy , D. J. Kelly , and F. J. O'Brien , Mater. Today Bio 3, 100009 (2019). 10.1016/j.mtbio.2019.100009 PMC7061547 32159148

[30] F. E. Freeman , H. Y. Stevens , P. Owens , R. E. Guldberg , and L. M. McNamara , Tissue Eng., Part A 22(19–20), 1176 (2016). 10.1089/ten.tea.2015.0339 27604384 PMC5073234

[31] J. Henkel , M. A. Woodruff , D. R. Epari , R. Steck , V. Glatt , I. C. Dickinson , P. F. Choong , M. A. Schuetz , and D. W. Hutmacher , Bone Res. 1(3), 216 (2013). 10.4248/BR201303002 26273505 PMC4472104

[32] M. Zhang , R. Lin , X. Wang , J. Xue , C. Deng , C. Feng , H. Zhuang , J. Ma , C. Qin , L. Wan , J. Chang , and C. Wu , Sci. Adv. 6(12), eaaz6725 (2020). 10.1126/sciadv.aaz6725 32219170 PMC7083611

[33] K. Inomata and M. Honda , Materials 12(18), 2869 (2019). 10.3390/ma12182869 PMC6765976 31491993

[34] X. Huang , B. Zhu , X. Wang , R. Xiao , and C. Wang , Int. J. Mol. Med. 38(4), 1141 (2016). 10.3892/ijmm.2016.2712 27571775 PMC5029969

[35] A. Papadimitropoulos , A. Scherberich , S. Guven , N. Theilgaard , H. J. Crooijmans , F. Santini , K. Scheffler , A. Zallone , and I. Martin , Eur. Cell Mater. 21, 445 (2011). 10.22203/eCM.v021a33 21604244

[36] Y. Kameo , Y. Miya , M. Hayashi , T. Nakashima , and T. Adachi , Sci. Adv. 6(10), eaax0938 (2020). 10.1126/sciadv.aax0938 32181336 PMC7060067

[37] H. Bahmaee , R. Owen , L. Boyle , C. M. Perrault , A. A. Garcia-Granada , G. C. Reilly , and F. Claeyssens , Front. Bioeng. Biotechnol. 8, 557111 (2020). 10.3389/fbioe.2020.557111 33015017 PMC7509430

[38] G. Cidonio , M. Cooke , M. Glinka , J. I. Dawson , L. Grover , and R. O. C. Oreffo , Mater. Today Bio 4, 100028 (2019). 10.1016/j.mtbio.2019.100028 PMC6894340 31853520

[39] D. G. Tamay , T. Dursun Usal , A. S. Alagoz , D. Yucel , N. Hasirci , and V. Hasirci , Front. Bioeng. Biotechnol. 7, 164 (2019). 10.3389/fbioe.2019.00164 31338366 PMC6629835

[40] A. S. Silva , L. F. Santos , M. C. Mendes , and J. F. Mano , Biomaterials 231, 119664 (2020). 10.1016/j.biomaterials.2019.119664 31855623

[41] O. H. Jeon , L. M. Panicker , Q. Lu , J. J. Chae , R. A. Feldman , and J. H. Elisseeff , Sci. Rep. 6, 26761 (2016). 10.1038/srep26761 27225733 PMC4881234

[42] Z. Chen , S. Ni , S. Han , R. Crawford , S. Lu , F. Wei , J. Chang , C. Wu , and Y. Xiao , Nanoscale 9(2), 706 (2017). 10.1039/C6NR06421C 27959374

[43] J. Whitehead , K. H. Griffin , M. Gionet-Gonzales , C. E. Vorwald , S. E. Cinque , and J. K. Leach , Biomaterials 269, 120607 (2021). 10.1016/j.biomaterials.2020.120607 33385687 PMC7870573

[44] Y. S. Kim , A. J. Chien , J. L. Guo , B. T. Smith , E. Watson , H. A. Pearce , G. L. Koons , A. M. Navara , J. Lam , D. W. Scott , K. J. Grande-Allen , and A. G. Mikos , J. Controlled Release 328, 710 (2020). 10.1016/j.jconrel.2020.09.048 PMC7749039 33010336

[45] J. Rauh , F. Milan , K. P. Gunther , and M. Stiehler , Tissue Eng., Part B 17(4), 263 (2011). 10.1089/ten.teb.2010.0612 21495897

[46] M. Hadida and D. Marchat , Biotechnol. Bioeng. 117(1), 251 (2020). 10.1002/bit.27171 31531968 PMC6915912

[47] C. F. Chen , Y. C. Chen , Y. S. Fu , S. W. Tsai , P. K. Wu , C. M. Chen , M. C. Chang , and W. M. Chen , Int. J. Mol. Sci. 22(16), 8987 (2021). 10.3390/ijms22168987 34445692 PMC8396436

[48] F. Chamieh , A. M. Collignon , B. R. Coyac , J. Lesieur , S. Ribes , J. Sadoine , A. Llorens , A. Nicoletti , D. Letourneur , M. L. Colombier , S. N. Nazhat , P. Bouchard , C. Chaussain , and G. Y. Rochefort , Sci. Rep. 6, 38814 (2016). 10.1038/srep38814 27934940 PMC5146967

[49] M. Zhu , W. Li , X. Dong , X. Yuan , A. C. Midgley , H. Chang , Y. Wang , H. Wang , K. Wang , P. X. Ma , H. Wang , and D. Kong , Nat. Commun. 10(1), 4620 (2019). 10.1038/s41467-019-12545-3 31604958 PMC6789018

[50] J. M. Sadowska , F. Wei , J. Guo , J. Guillem-Marti , Z. Lin , M. P. Ginebra , and Y. Xiao , Acta Biomater. 96, 605 (2019). 10.1016/j.actbio.2019.06.057 31269454

[51] A. Anitha , D. Menon , T. B. Sivanarayanan , M. Koyakutty , C. C. Mohan , S. V. Nair , and M. B. Nair , ACS Appl. Mater. Interfaces 9(32), 26707 (2017). 10.1021/acsami.7b07131 28741921

[52] Y. S. Kim , M. Majid , A. J. Melchiorri , and A. G. Mikos , Bioeng. Transl. Med. 4(1), 83 (2019). 10.1002/btm2.10110 30680321 PMC6336671

[53] T. N. Vo , F. K. Kasper , and A. G. Mikos , Adv. Drug Delivery Rev. 64(12), 1292 (2012). 10.1016/j.addr.2012.01.016 PMC3358582 22342771

[54] A. Marrella , T. Y. Lee , D. H. Lee , S. Karuthedom , D. Syla , A. Chawla , A. Khademhosseini , and H. L. Jang , Mater. Today 21(4), 362 (2018). 10.1016/j.mattod.2017.10.005 PMC6082025 30100812

[55] X. Cun and L. Hosta-Rigau , Nanomaterials 10(10), 2070 (2020). 10.3390/nano10102070 PMC7590059 33092104

[56] K. Metavarayuth , P. Sitasuwan , X. Zhao , Y. Lin , and Q. Wang , ACS Biomater. Sci. Eng. 2(2), 142 (2016); 10.1021/acsbiomaterials.5b00377 33418629

[57] Y. S. Torisawa , C. S. Spina , T. Mammoto , A. Mammoto , J. C. Weaver , T. Tat , J. J. Collins , and D. E. Ingber , Nat. Methods 11(6), 663 (2014). 10.1038/nmeth.2938 24793454

[58] J. Chen , M. Hendriks , A. Chatzis , S. K. Ramasamy , and A. P. Kusumbe , J. Bone Miner. Res. 35(11), 2103 (2020). 10.1002/jbmr.4171 32845550

[59] F. Yu , W. Hunziker , and D. Choudhury , Micromachines 10(3), 165 (2019). 10.3390/mi10030165 PMC6470849 30818801

[60] J. R. Garcia and A. J. Garcia , Drug Delivery Transl. Res. 6(2), 77 (2016). 10.1007/s13346-015-0236-0 PMC4662653 26014967

[61] T. Tian , T. Zhang , Y. Lin , and X. Cai , J. Dent. Res. 97(9), 969 (2018). 10.1177/0022034518767120 29608865

[62] Y. Yan , H. Chen , H. Zhang , C. Guo , K. Yang , K. Chen , R. Cheng , N. Qian , N. Sandler , Y. S. Zhang , H. Shen , J. Qi , W. Cui , and L. Deng , Biomaterials 190–191, 97 (2019). 10.1016/j.biomaterials.2018.10.033 30415019

[63] Y. Shanjani , Y. Kang , L. Zarnescu , A. K. Ellerbee Bowden , J. T. Koh , D. F. E. Ker , and Y. Yang , J. Mech. Behav. Biomed. Mater. 65, 356 (2017). 10.1016/j.jmbbm.2016.08.037 27631173

[64] R. M. da Silva , J. F. Mano , and R. L. Reis , Trends Biotechnol. 25(12), 577 (2007). 10.1016/j.tibtech.2007.08.014 17997178

[65] C. Hu , Y. Chen , M. J. A. Tan , K. Ren , and H. Wu , Analyst 144(15), 4461 (2019). 10.1039/C9AN00421A 31162494

[66] X. Wang , Q. Sun , and J. Pei , Micromachines 9(10), 493 (2018). 10.3390/mi9100493 PMC6215090 30424426

[67] M. Zaidi , T. Yuen , L. Sun , and C. J. Rosen , Endocr. Rev. 39(5), 701 (2018). 10.1210/er.2018-00050 29897433 PMC6173473

[68] Z. Lin , D. Shen , W. Zhou , Y. Zheng , T. Kong , X. Liu , S. Wu , P. K. Chu , Y. Zhao , J. Wu , K. M. C. Cheung , and K. W. K. Yeung , Bioact. Mater. 6(8), 2315 (2021). 10.1016/j.bioactmat.2021.01.018 33553818 PMC7840811

[69] P. Humbert , M. A. Brennan , N. Davison , P. Rosset , V. Trichet , F. Blanchard , and P. Layrolle , Front. Immunol. 10, 663 (2019). 10.3389/fimmu.2019.00663 31001270 PMC6455214

[70] S. Nadine , C. R. Correia , and J. F. Mano , Adv. Healthcare Mater. 10(10), e2001993 (2021). 10.1002/adhm.202001993 33506631

[71] X. Zhang , Q. Chen , and X. Mao , BioMed Res. Int. 2019, 7908205. 10.1155/2019/7908205 31828131 PMC6885163

[72] D. P. Vasconcelos , A. P. Aguas , M. A. Barbosa , P. Pelegrin , and J. N. Barbosa , Acta Biomater. 83, 1 (2019). 10.1016/j.actbio.2018.09.056 30273748

[73] F. Khan and M. Tanaka , Int. J. Mol. Sci. 19(1), 17 (2017). 10.3390/ijms19010017 PMC5795968 29267207

[74] D. M. Vasconcelos , R. M. Goncalves , C. R. Almeida , I. O. Pereira , M. I. Oliveira , N. Neves , A. M. Silva , A. C. Ribeiro , C. Cunha , A. R. Almeida , C. C. Ribeiro , A. M. Gil , E. Seebach , K. L. Kynast , W. Richter , M. Lamghari , S. G. Santos , and M. A. Barbosa , Biomaterials 111, 163 (2016). 10.1016/j.biomaterials.2016.10.004 27728815

[75] J. E. Won , Y. S. Lee , J. H. Park , J. H. Lee , Y. S. Shin , C. H. Kim , J. C. Knowles , and H. W. Kim , Biomaterials 227, 119548 (2020). 10.1016/j.biomaterials.2019.119548 31670033

[76] T. U. Luu , S. C. Gott , B. W. Woo , M. P. Rao , and W. F. Liu , ACS Appl. Mater. Interfaces 7(51), 28665 (2015). 10.1021/acsami.5b10589 26605491 PMC4797644

